# Three dimension high definition manometry evaluated postoperative anal canal functions in children with congenital anorectal malformations

**DOI:** 10.3389/fped.2023.1126373

**Published:** 2023-06-16

**Authors:** Jiawei Zhao, Yanan Zhang, Yu Xiong, Jingbin Du, Yongwei Chen, Weihong Guo, Jinshi Huang

**Affiliations:** Department of Neonatal Surgery, Beijing Children’s Hospital, Capital Medical University, National Center for Children’s Health, Beijing, China

**Keywords:** anorectal manometry, anorectal malformations, anorectal function, postoperative complications, prognosis

## Abstract

**Background:**

We aimed to evaluate the function of the reconstructed anal canal in postoperative anorectal malformations (ARMs) patients through three dimension (3D) high-definition anorectal manometry.

**Methods:**

From January 2015 to December 2019, 3D manometry was performed as a postoperative functional assessment of patients with ARMs divided into age subgroups based on the time of manometry. Manometric parameters, such as the length of the anorectal high-pressure zone (HPZ-length), the mean resting and squeeze pressure of HPZ (HPZ-rest and HPZ-sqze), recto-anal inhibitory reflex (RAIR), and strength distribution of the anal canal, were collected and compared with age-matched controls. Their functional outcomes were analyzed with SPSS 23.0 software for statistical analysis.

**Results:**

171 manometric measurements were performed on 142 postoperative patients (3 months∼15 years). The HPZ-rest in all patients was significantly lower than in age-matched controls (*p *< 0.05). HPZ-sqze was notably decreased in patients older than 4 years, whereas other age groups were comparable to controls (*p *< 0.05). The proportions of asymmetric strength distribution and negative RAIR were higher in ARMs patients. The type of anorectal malformations and lower HPZ-rest were the impact factors affecting postoperative functional outcomes.

**Conclusions:**

The majority of the ARMs patients had acceptable functional outcomes. 3D manometry can objectively assess the reconstructed anal canal function. The patients with fecal incontinence had a high proportion of extremely low HPZ-rest and HPZ-sqze, negative RAIR, and asymmetric strength distribution. The manometric details will help the clinicians explore the causes of defecation complications and guide further management.

## 1. Introduction

Congenital anorectal malformations (ARMs) are the most commonly seen developmental malformation of the digestive tract, with an incidence in China was 3.17 cases per 10,000 populations, according to the data collected from the Chinese Birth Defects Monitoring Network (1). Surgical management is required primarily soon after birth or in early infancy. Whether the reconstructed anus would have similar defecation as ordinary people is the most critical concern not only for the parents but also for the surgeons. Constipation and fecal incontinence are commonly seen as long-term complications. The postoperative function is usually assessed by the conventional clinical scoring system based on subjective parameters such as the number of defecation and the ability to have voluntary bowel movements (2, 3). Recently, three dimension (3D) high definition anorectal manometry has been believed to be a precise tool for assessing defecation function because it can provide quantitative, objective measurements in real-time and dynamically (4, 5). Compared to the water-perfused manometry system, 3D manometry offers not only in terms of imagining but also an understanding of the functional and potential pathophysiology of functional anorectal disorders. There was a study comparing the fecal continence scores of patients with ARMs with anorectal manometric findings (6). Our present study aimed to use 3D manometry to evaluate anal function in ARMs patients and controls so that more detailed and valuable references can be provided. This might help physicians better understand postoperative anal function and find the true reasons for complications.

## 2. Materials and methods

### 2.1. Patients

We reviewed all patients associated with ARMs who were admitted to Beijing Children's Hospital from January 2015 to December 2019. One hundred seventy-one manometry measurements were performed on 142 postoperative patients who were followed up at the Beijing Children's Hospital outpatient clinic. Among them, 132 patients underwent surgery in our hospital, and routine follow-up began 3 months after surgery, whether having complications or not. The other 10 patients had surgery in other hospitals but came to our clinic because of poor bowel function. A manometric exam was repeated in those with poor bowel function when the patient grew up or after conservative management. During the same period, 71 children from our previous study were selected as the age-matched non-operative control groups (7). The selection criteria of this group were as follows: children without the organic anorectal disease (e.g., Hirschsprung's disease) identified by imaging and rectal mucosal biopsy; children without diagnosed functional constipation; children without spinal or vertebral deformity; children without anorectal or spinal surgery. These groups of children who served as controls were confirmed to have regular bowel habits during the follow-up. Manometry measurements were conducted following the physical examinations at the outpatient clinic. The following manometric parameters were collected, such as the length of the anorectal high-pressure zone (HPZ-length), the mean resting and squeeze pressure of HPZ (HPZ-rest and HPZ-sqze), recto-anal inhibitory reflex (RAIR), and strength distribution of the anal canal. Information on malformation and surgical management was obtained through the electronic medical record system.

Postoperative outcomes were graded as good, fair, and poor according to their bowel movements. Good was defined as patients with regular bowel movements without any defecation complaints. Fair was defined as complaints of constipation or soiling, and constipation could be resolved with the help of oral medication or laxatives, whereas dirty pants occasionally occurred under the situation of sleeping, coughing, and diarrhea. Poor was defined as severe constipation in that bowel management (e.g., enema) was needed, whereas incontinence was defined as a recurrent involuntary loss of liquid and/or solid stool in the older child and frequent dirty pants in toddlers. Based on the age at examination, patients were divided into three groups (3–12 months, 1–4 years, and >4 years). The basis of age grouping is quoted from our previous study and the others (7–9). This study was approved by the Medical Ethics Committee of Beijing Children's Hospital (2018-K-129), and the requirement for informed patient consent was waived.

### 2.2. Anorectal manometry

**Figure 1:**
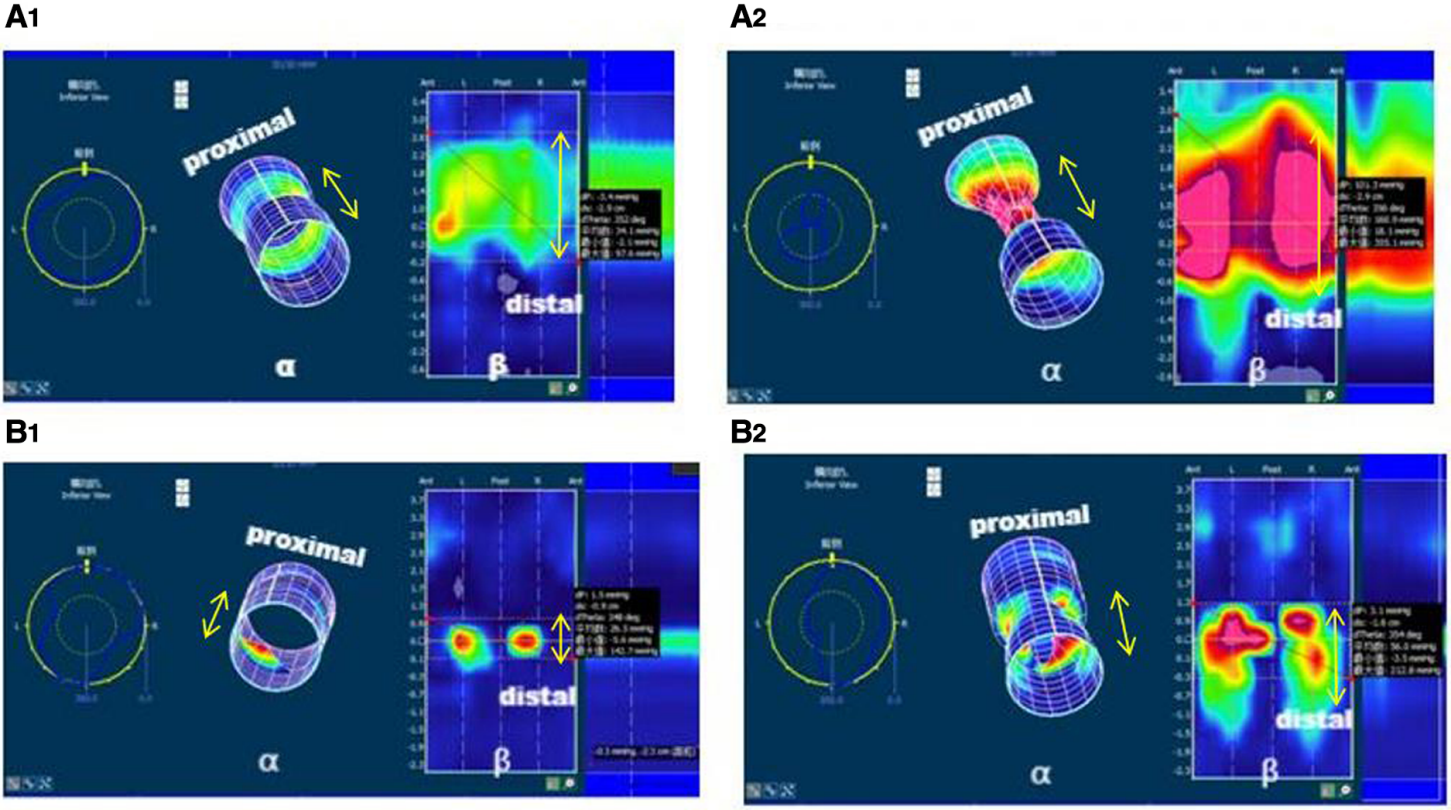
3D columnar and plane pressure map of anal tube strength distribution. β was the 2D rectangle grid plane pressure map when the 3D column (α) was cut along the anterior midline so that 4 quadrants (anterior, posterior, right, and left) were divided. The double arrow indicated the length of high-pressure zone. Patient A, 1 year 11 months, cloaca, postoperative 18 months with occasional constipation. Normal HPZ-length, HPZ-rest, and HPZ-sqze with symmetric strength distribution in the resting phase (A1) and squeeze phase (A2). Patient B, 7 years, cloaca, postoperative 72 months with severe fecal incontinence. Short HPZ-length, significantly lower HPZ-rest, and HPZ-sqze with asymmetric strength distribution in resting (B1, lacking in both posterior and anterior) and squeeze phase (B2, lacking in both posterior and anterior).

The measurement of anal canal function was performed with a high-definition manometry catheter (ManoScan 360HD, Sierra Scientific Instruments, Los Angeles, California). The mean pressure of the anal area was calculated with the SmartmouseTM and the implemented software algorithm (ManoView analysis software, Sierra Scientific Instruments, Los Angeles, California). All procedures were performed by the same registered nurse and analyzed by 2 senior physicians. The patient was put into the supine or lateral position. Physicians inserted a lubricated balloon catheter (10.75 mm in diameter, 6.4 cm in length equipped with 256 pressure sensors in 16 rows and 16 circumferentially oriented) in the anus and calibrated pressure to the baseline position. Patients younger than 4 years old who could not complete the examination required sedation with chloral hydrate. (1) Inserted the catheter into the anus for about 10 cm, observed for 1–2 min in a calm state, and measured the HPZ-length and HPZ-rest; (2) Patients under 4 years older were stimulated by perianal pinprick, whereas those older than 4 years were asked to contract voluntarily to measure HPZ-sqze; (3) RAIR was evaluated by rapid inflation and deflation of the balloon with 10-ml incremental volumes ranging from 10 to 60 ml. The reflex was considered present when the decrease reached 25% of the resting pressure. HPZ-length, HPZ-rest, and HPZ-sqze were measured three times with a 30-second break. As shown in Figure 1, the 3D column diagram (α) could be cut along the anterior line and unfolded into a 2D rectangle grid (β) so that the anal canal could be easily recognized as 4 quadrants (anterior, posterior, left, and right). The anal pressure and strength distribution could be viewed and calculated in 2D topographical color plots of all pressure transducers. When the proximal and distal ends of the high-pressure zone were established, the length and the mean pressure were calculated with the aid of the Smartmouse™ and the implemented software algorithm. Detailed anorectal manometry procedures can be quoted from our previous study (7).

### 2.3. Statistical methods

All analyses were performed using SPSS, version 23.0 (IBM Corp, Armonk, NY), and a *p*-value of less than 0.05 was considered statistically significant. Numerical variable data were presented as a mean ± standard deviation, and group comparisons were performed using the Student's *t*-test. Categorical variable data was described by rate or proportion, and group comparisons were performed by *χ*^2^ or Fisher test. Correlation analysis was conducted with Pearson or Spearman correlation coefficient depending on data distribution. Logistic regression analysis was used for analyzing the influencing factors of postoperative anal function.

## 3. Results

### 3.1. Patient characteristics

One hundred forty-two patients (61 boys and 81 girls) were included in the surgery group, ranging from 3 months to 15 years (median 1.71 years). The ARMs were classified into high (10%), intermediate (28%), and low-type (62%) by Wingspread classification based on the level of the rectum in relation to the levator ani muscle (10, 11). Types of fistula category included no fistula (1%), anocutaneous fistula (42%), rectovestibular fistula (25%), rectobulbar-urethral fistula (17%), rectoprostatoc-urethral fistula and rectovesical fistula (5%), rectovaginal fistula (6%), cloaca (4%). Among the 142 patients, 40 (28%) cases underwent staged surgery with posterior sagittal anorectoplasty or laparoscopically assisted anorectal pull-through. The other 102 cases with anocutaneous fistula and rectovestibular fistula were treated with direct sagittal or anterior sagittal anorectoplasty. Eighty percent of these patients had socially acceptable postoperative fecal continence, with functional outcomes graded as good at 50% and fair at 30%. Within the 20% of patients with poor functional outcomes, cases of fecal incontinence were more than severe constipation (Table 1).

**Table 1:** Characteristics of 142 ARMs patients and postoperative functional outcomes.

	Good (*n* = 72)	Fair (*n* = 42)		Poor (*n* = 28)	
		Constipation (*n* = 20)	Soiling (*n* = 22)	Severe constipation (*n* = 8)	Incontinence (*n* = 20)
male: female	32:44	7:13	10:12	3:5	9:11
Low					
Without fistula	1	0	0	0	1
Anocutaneous fistula	34	11	10	2	3
Anovestibular fistula	17	3	3	2	1
Intermediate					
Without fistula	0	0	0	0	0
Rectobulbar-urethral fistula	12	1	5	1	5
Rectovestibular fistula	2	1	1	3	3
Rectovaginal fistula	3	2	0	0	1
High					
Without fistula	0	0	0	0	0
Rectoprostatic-urethral fistula/Rectovesical fistula	2	0	2	0	3
Rectovaginal fistula	0	0	1	0	1
Cloaca	1	2	0	0	2
Associated spinal/vertebral deformity	10	6	4	5	6

Good was defined as regular bowel movement. Fair was defined as constipation but could be solved by oral medication or laxatives, whereas soiling was defined as occasional dirty pants occurred during sleep, cough, and diarrhea. Poor was defined as severe constipation that bowel management (e.g., enema) was needed, whereas incontinence was defined as a recurrent involuntary loss of liquid and/or solid stool in older child and frequent dirty pants in toddlers.

Surgical management was classified into 3 approaches according to the operation procedure and dissection of the sphincter ring. In a low imperforated anus without fistula and staged laparoscopy-assisted anorectoplasty, the incision and pull-through at the center of the anal dimple allow it only separates parasagittal fibers and muscle in the mid-line so that the sphincter ring was preserved intact, while in posterior and anterior anorectoplasty, neither the anterior nor posterior muscle complex was intact.

We have studied the relationship between poor functional outcomes and the classification of ARM, type of fistula, and surgical approaches. Statistical analysis showed poor outcomes related to the classification of high position and cloaca (OR = 4.821, *p* = 0.011) but not the types of fistula and surgical approaches.

### 3.2. Anorectal manometry of the anorectal canal

#### 3.2.1. Comparison between patients and controls

Manometric measurements of the reconstructed anal canal were collected and compared with their age-matched non-operative controls. Significantly lower HPZ-rest was seen in all postoperative groups (*p *< 0.05), though there was no apparent difference in the HPZ-length. The HPZ-sqze in the >4 years group was remarkably lower than its control, whereas the other age groups were comparable (Table 2).

**Table 2:** Comparison of basic manometric parameters between ARMs patients and non-operative controls.

Age Group	Number	HPZ-length		*p*-value	HPZ-rest		*p*-value	HPZ-sqze		*p*-value
	patients:controls	patients	controls		patients	controls		patients	controls	
3–12 months	30:16	2.48 ± 0.46	2.32 ± 0.34	0.172	32.72 ± 12.30	43.94 ± 14.86	0.002*	108.11 ± 35.35	114.59 ± 34.60	0.525
1–4 years	82:24	2.65 ± 0.52	2.45 ± 0.42	0.099	28.08 ± 10.54	43.05 ± 11.12	<0.001*	99.78 ± 35.13	107.90 ± 45.30	0.410
>4 years	30:31	2.69 ± 0.60	2.64 ± 0.50	0.713	28.38 ± 11.90	55.97 ± 12.72	<0.001*	91.04 ± 29.74	141.46 ± 35.69	<0.001*
Total	142:71	2.57 ± 0.51	2.50 ± 0.45	0.307	30.43 ± 11.80	48.89 ± 14.03	<0.001*	102.61 ± 34.84	125.09 ± 41.21	<0.001*

HPZ-length, the functional length of anorectal high-pressure zone; HPZ-rest, resting pressure of high-pressure zone; HPZ-sqze, squeeze pressure of high-pressure zone. Data was reported as mean ± standard deviation.

* The difference was statistically significant.

**Figure 2:**
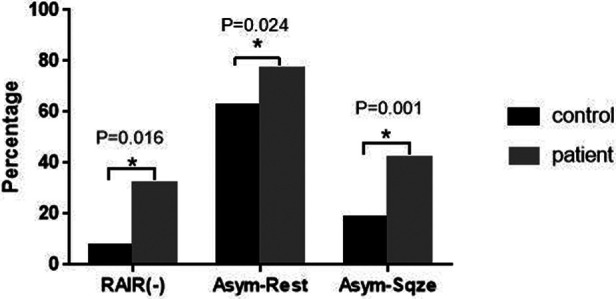
Statistic results of RAIR (-) and asymmetric strength distribution between ARMs patients and controls. Asym-Rest: rate of asymmetric strength distribution at resting phase. Asym-Sqze: rate of asymmetric strength distribution at squeezing phase.*p < 0.05.

Negative RAIR was found in 32% (46 cases) of patients and was significantly higher than the 7% (5 cases) in the controls (*p *< 0.05). Most of these negative patients had regular bowel movements or constipation, and few had severe constipation symptoms. Contrast angiography and biopsies were performed, and Hirchsprung's disease was confirmed only in one rectovestibular fistula patient with recurrent abdominal distension and severe constipation. Pull-through surgery was performed eventually. The 2D and 3D surface color plots of the anal canal revealed that the asymmetric strength distribution in patients had a significantly higher percentage in the resting and squeezed phases than in the controls (Figure 2).

#### 3.2.2. Comparison of ARMs patients between good and poor bowel function

**Figure 3:**
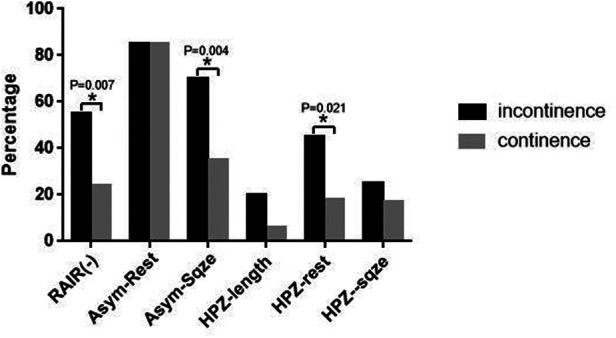
Statistic results of comparing the patient number of the poor manometric parameter in ARMs between incontinence and continence group. *p < 0.05.

Of the 28 patients with poor functional outcomes, 20 had fecal incontinence, while 8 had severe constipation. Of the 42 patients with fair function, 20 had frequent constipation but could be resolved by oral medication or laxatives, while the other 22 had soiling only under certain situations such as sleeping, coughing, and diarrhea. The remaining 72 patients had good bowel function (Table 1). There were only 8 cases of severe constipation, and it was hard to draw any statistical conclusion considering the sample size between the constipation groups. Our present study focused on 20 patients with fecal incontinence. The manometric parameters were compared with their age-grouped mean ± SD. A significantly lower measurement was noticed when the value was lower than its mean-SD. The percentages of these patients were counted and compared between the 20 incontinence and 94 continence patients (not including constipation). Statistic studies showed that the majority of incontinence patients had significantly lower HPZ-rest (*p *= 0.021, Table 3). Meanwhile, 70% (14 cases) of this group of patients had an asymmetric distribution of the anal canal seen in the squeeze phase comparing the 35% (33 cases) in the continence group (*p *= 0.004). Moreover, 55% (11 cases) of patients presented negative RAIR was also significantly higher than 24% (23 cases) of the continence group (*p *= 0.007, Figure 3).

**Table 3:** Comparison of the patient number of the poor manometric parameter in ARMs between incontinence and continence group.

Defecation function	HPZ-length		*p*-value	HPZ-rest		*p*-value	HPZ-sqze		*p*-value
	<mean-SD	≥mean-SD		<mean-SD	≥mean-SD		<mean-SD	≥mean-SD	
Incontinence (*n* = 20)	4	16	0.129	9	11	0.021*	5	15	0.604
Continence (*n* = 94)	6	88		17	77		16	78	

Continence: patients classified as good + soiling. Data was reported as patient numbers of each manometric parameter. <mean-SD represented manometric measurement was lower than their age-grouped mean-SD, ≥mean-SD represented manometric measurement was equal or greater than their age-grouped mean-SD.

* The difference was statistically significant.

Considering the classification of ARM, type of fistula, surgical approach, and anal functional parameters might be the impact factors that influence the poor outcome. The logistics regression study showed that the classification of ARMs (high and cloaca, OR = 4.821, *p* = 0.011) and lower HPZ-rest (OR = 3.767, *p* = 0.033) were the two impact factors associated with poor postoperative functional outcomes.

## 4. Discussion

Congenital anorectal malformations are the most commonly seen congenital disease anatomically manifested as anal canal developmental malformations at the end of the digestive tract. Defecation can only be achieved through surgical reconstruction of the anal canal. Common postoperative long-term complications include constipation and fecal incontinence (12). Usually, bowel function was evaluated by various scoring systems based on questionnaires focusing on postoperative daily bowel movements and voluntary defecation control. However, the defecation patterns result from bowel movements under a combination of many factors involved in the mechanism. Thus the scoring system can not reveal the exact functional ability of the reconstructed anal canal and its role in defecation and continence.

The anorectal manometric study is well established for quantifying anal sphincter tone, assessing the anorectal sensory response, reflexes, strength distribution, and rectal compliance quantitatively. It is also crucial for evaluating restorative surgical procedures of the anorectal area for adults and children, with the advantages of being non-invasive, safe, and intuitive. Compared with water-perfused manometry systems, developing solid catheters with sensors has enabled precise measurement (high definition) and the creation of anorectal 3D pressure models, making it possible to visualize and localize a potential defect in the structure of the internal sphincter. This method offers more accurate and detailed data that can be employed in assessing functional disorders.

Most previous studies focus on evaluating the postoperative anal function between different types of ARMs or different surgical managements without comparing it with the non-operative control group (13, 14). Since most surgical managements were completed within infancy, the rapid growth of the physical anal canal during the newborn and infantile period should significantly affect the functional anal canal. Our previous study (7) revealed that age is essential when evaluating anal canal function in non-operative children. The present study was designed based on age to evaluate the functional parameters of the reconstructed anal canal in patients with ARMs.

Since most of our patients were routinely followed up at the scheduled time, the manometric parameters collected could reflect the general situation of the anal canal after reconstruction surgery. Functional anal canal length is the area over which the resting anal pressures exceed the resting intrarectal pressure by at least 5 mmHg. The length of the high-pressure zone is rather a functional but not an anatomic structure. Approximately 50%–85% of the pressure in the resting phase is of internal anal sphincter (IAS) origin, whereas in the squeeze phase is the combined effect of effect of external anal sphincter (EAS) and puborectalis contraction. These sphincter complexes maintaining the high-pressure zone can explain why the HPZ-length of the reconstructed anal canal was comparable with the non-operative control. Since resting pressure serves as a measurement of IAS function, the significantly lower HPZ-rest in all surgery patients than that of their age-matched controls indicated a weakness or degeneration of IAS (Table 2). Most children aged >4 years could contract the anus voluntarily, and the pressure represents the maximum voluntary squeeze pressure of the anal canal. In infants and toddlers younger than 4, perianal pinprick was used to induce passive anal contractions under sedation. This test was more like a cough reflex performed in the adult during the manometric examination that reflected the natural contraction ability of EAS. Though the passive anal contractions under sedation did not represent the muscles that could be controlled voluntarily, it might prove whether the EAS has the ability to contract and the normal existence of the local reflex arc between the EAS and the spinal cord. Weakness or damage of the EAS or local nerve should be considered if the patient had significantly lower HPZ-sqze compared to its age-matched control. The squeeze maneuver needs to be repeated when the patients grow old enough if the incontinence persists. The HPZ-sqze in two of the younger age groups were comparable with their controls, indicating the similar function of EAS. Older patients came to clinical because of poor functional outcomes. This might be explained why the >4 years group had significantly lower HPZ-sqze than its control.

The surgical management during anal canal reconstruction might also affect muscles and nerves locally, no matter what procedure was performed. Correct placement of the rectum within the center of the muscle complex is critical for the optimal functional outcome. Our statistical analysis showed that functional outcome was correlated only with the classification of the ARMs but not the surgical approaches. This indicated that the development of sphincter muscle complexes and nerve networks might be the dominant reasons. For those incontinence ARMs patients who were not classified as high-type, besides the abnormal embryonic development of the sphincter and nerve system, other reasons should be considered, such as massive dissection during the surgical procedure or the wrong position of the anal tract being placed so that the external sphincters did not appropriately surround it during the reconstruction.

With 3D models, our present results showed that even in the non-operative controls, the strength distribution of the anal canal was not as symmetrical as we imagined. This might be due to the individually different development of each muscle in the sphincter complex involved in maintaining the high-pressure zone. Similar results were seen in the majority of the ARMs patients that had acceptable postoperative bowel function. In those ARMs patients with poor incontinence, the 3D asymmetric strength distribution could help to locate the weak area so that further image studies such as MRI (15) or ultrasound (16) might be focused on identifying the particular muscle complexes.

RAIR plays a prominent role in normal defecation and sensation. The mechanism of RAIR and the neural pathway of reflex remained unknown. It is believed that the RAIR mainly depends on the intrinsic intramural nerve network regulated by the sacral medullary (17). In a systematic review, Rajasegaran (18) summarized several papers and concluded that the absence of RAIR in the poor continence group might indicate low quality or damage of the IAS, which may already be poorly developed in patients with ARMs. Similar results were seen in our present study, with a higher incidence of negative RAIR and a significantly lower HPZ-rest in the incontinence patients, suggesting the poor sensation and weakness of their IAS. Negative RAIR is often seen in HD. Though ARMs combined with HD were rare, accounting for only about 2% based on the previous study (19), the only one combined with HD case in our study reminds the importance of biopsy ruling out HD.

About 20% of our patients were graded as fecal incontinence. These were the group of patients we were more concerned about their manometric measurements. For those whose manometric parameters were comparable to their age-matched controls, other reasons were founded, such as some had associated with spinal deformity, and even an infective rectovestibular fistula was found in a patient who had her definite surgery in the other hospital. For those with poor manometric measurements, conservative treatments were prescribed, including dietary fiber supplementation, stool-modifying drugs, biofeedback physiotherapy, trans-anal irrigation, or combinations thereof. A follow-up manometric examination was performed after a certain period of treatment so that the manometric parameters were collected and compared to see if any of them could be improved.

However, the patients in our study were relatively young, and few patients could complete defecation perception and other tests, which was a limitation of this study. In addition, all patients and controls in this study used the same manometry equipment, and manometry procedure, operated by the same nurse, and analyzed the results by the same senior physicians. Confounding factors were excluded as much as possible within our ability, and the manometry data obtained could be used for reference objectively.

## 5. Conclusions

To conclude our study, age should be considered an important factor when evaluating postoperative function in ARMs patients. The classification of high and cloaca in ARMs and lower HPZ-rest were two factors that affected postoperative function. Different operative procedures or approaches would not affect the functional outcome. 3D high definition manometry is a valuable modality providing accurate details of anal function. The proper assessment will provide physicians with information on potential causes of complications. Meanwhile, it will also provide the selection of an adequate treatment modality.

## Data Availability

The original contributions presented in the study are included in the article, further inquiries can be directed to the corresponding authors.
